# Bioethical considerations for cancer patients care during the COVID-19 pandemic

**DOI:** 10.2217/fon-2020-0742

**Published:** 2020-09-09

**Authors:** Hampig Raphael Kourie, Roland Eid, Fady Gh Haddad, Michel Scheuer, Roland Tomb

**Affiliations:** ^1^Department of Hematology-Oncology, Faculty of Medicine, Saint Joseph University of Beirut, Riad El Solh, 11-5076, Lebanon; ^2^Ethical Committee of Saint Joseph University of Beirut, Riad El Solh, 11-5076, Lebanon; ^3^Department of Dermatology, Faculty of Medicine, Saint Joseph University of Beirut, Riad El Solh, 11-5076, Lebanon

**Keywords:** bioethical, bioethics, COVID-19, medical ethics, outbreak, pandemic, principles

SARS-Cov-2, the novel coronavirus responsible for COVID-19, has become a global threat and a healthcare concern. Human-to-human transmission of the virus occurs through respiratory droplets (by coughing or sneezing) and through direct contact with an infected patient or indirect contact with fomites in their environment.

Cancer patients are known to be more vulnerable and fragile toward viruses with an increased risk of hospitalizations and deaths compared with the general population [1]. According to recently published Chinese data, there is no increase in the incidence of COVID-19 infection in cancer patients; however, cancer patients had a higher incidence of serious events (hospitalization, respiratory complications and intensive care unit [ICU] admission) compared with non-cancer patients [2].

The four principles initiated and popularized by Beauchamp and Childress [3]—autonomy, nonmaleficence, beneficence and justice—are not considered universal can openers, but they are useful for guiding our reflection on the current approach to ethics of care for cancer patients during the COVID-19 pandemic. Previous studies have discussed different aspects related to clinical research ethics such as nonabandonment, flattening of the curve and emotional support, which are considered essential for the cancer research community [4]. On a daily basis, physicians and healthcare workers who treat cancer patients infected with COVID-19 are subject to ethical challenges. In this editorial, we aim to highlight bioethical considerations in the management of cancer patients during the COVID-19 pandemic based on the four principles of bioethics.

## Autonomy

During pandemics, the authorities intervene in the daily life of their citizens by limiting or prohibiting certain activities or by forcing them to do others; personal freedom will be preceded by the general interest. All patients aware of their SARS-CoV-2 infection should report and take the necessary measures to avoid infecting other patients by quarantining themselves. If they refuse, the authorities have the right to compel them to do so. The autonomy of cancer patients during the pandemic will take other aspects, because their priority and potentially fatal risk is theoretically more linked to their disease than to the virus itself. Cancer patients during this COVID-19 pandemic have the right to decide, in agreement with their treating physician, how to manage and deal with their disease by continuing their treatment, withholding it or stopping it. The fear and anxiety associated with possible SARS-CoV-2 infection could influence the cancer patients’ decision regarding their treatment. The role of the oncologist is to explain the advantages and disadvantages of continuing or stopping the treatment depending on the characteristics of the patient and the stage and nature of the disease. In this perspective and in front of this complex and stressful situation, the healthcare team can help the patient make his decision and in particular encourage him to speak about it very openly with his spouse, his children or his trusted person while clearly recognizing that the final decision is certainly up to him.

## Nonmaleficence & beneficence

The principles of nonmaleficence and beneficence are questioned in the treatment of cancer patients during the COVID-19 pandemic: will anti-cancer drug treatment be beneficial or harmful for cancer patients? According to some preliminary data from China, certain cancer treatments such as chemotherapy and immunotherapy are likely to increase the risk of serious complications due to SARS-CoV-2 infection [2]. Moreover, it is well known that chemotherapy can compromise the immune system of cancer patients and increase their risk of infection. However, refraining from treating a patient with cancer during this pandemic will relatively reduce their chance of curing or stabilizing their cancer. Oncologists face a big dilemma between treating their patients with all of the possible risks and complications of COVID-19 and withholding therapy to save their patients from the infectious threat while risking a possible increase in the risk of metastases, relapse or cancer progression. It is therefore important to categorize cancer patients according to the stage of their disease and the treatment resulting from it (curative vs palliative). On one hand, patients with a high chance of cure and a long life expectancy should be advised to continue their treatment, despite the risk of infection and its complications, especially when the benefit of this treatment is widely proven. However, in these cases, it is necessary to maximize the protective measures within the hospital to avoid the infectious risks secondary to the admission of patients and therefore respect the principle of nonmaleficence. On the other hand, people with advanced disease, in whom postponing or skipping certain treatment sessions does not affect cancer-related survival but reduces the risk of infection by limiting exposure to hospital and clinic visits, should avoid receiving these therapies during the crisis period. At the same time, we must not forget to remind them how to optimize home precautions, by teaching them to avoid contact and to respect preventive hygienic recommendations.

## Priority & justice

No human life has more or less value than another. This must be the very first guide for caregivers. Actually, the COVID-19 pandemic has suddenly elucidated an imbalance between medical needs and the available resources in health management. A high rate of admissions and transfers to intensive care units has been observed, risking the flooding of COVID-19-infected cases. Faced with these high demands, the decision to accept patients in ICUs remains critical. The prioritization of admissions to ICUs is often adapted in some countries to overcome the flow of patients requiring an admission to these units. Consequently, physicians are obliged to choose and select the patients who will benefit from a mechanical ventilator. The patient ‘triage’ process represents a big dilemma for critical care physicians during this pandemic. In previous health disasters, medical authorities were confronted with the concept of prioritization in healthcare; one of the major criteria for ‘sorting patients’ was considered to be the patient’s lifespan more than his age. Lifespan was linked to the presence of comorbidities, including cancer. Any discrimination based on wealth, ethnicity, nationality or social status was and should be prohibited, especially in countries where the financial capacity of patients and their insurability are most uneven. The prioritization of medical staff infected with COVID-19 may be justified, as they have exposed their life and health only to take care of others. Cancer patients being more vulnerable could be discriminated by certain physicians because of their illness and lifespan, and other non-cancer patients could be prioritized. The justice/equity between cancer patients and the general population must be respected during the COVID-19 pandemic, and discrimination against these patients should not be systematically considered. In this perspective, if the hospital establishment has an ethics committee, consultation of this committee in the event that ‘sorting’ of patients is necessary should be compulsory. It is indeed a consultation, the decision remaining in the hands of the doctor in charge. That being said, cancer patients receiving treatment with curative intent generally have a very good life expectancy comparable to the general population. Therefore, ‘having cancer’ should not be a discriminating factor in this case. Meanwhile, for cancer patients with end-stage disease and terminal illness, whose life expectancy is limited to few weeks or months, oncologists should clearly explain to them and to their families the inutility of a transfer to an ICU, without letting them feel neglected or less important compared with other patients.

## Ethical & equal access to care

Every life has equal worth, and it is an ethical imperative to assure adequate care for developed and underdeveloped populations across the world. Despite being an ethical obligation, it is a common interest for all countries to secure global allocation for scarce resources to address the COVID-19 outbreak in one part of the world in order to help prevent a surge of the infection in the rest of the world. However, testing and other medical resources needed for the management of the pandemic are lacking in poor counties, whereas privileged richer countries take precedence in their acquisition. Giant economies throughout the world should take short-term and medium-term actions to help governments with less advanced economies. Coordinated programs and pooling regional procurement via large international organization (e.g., the WHO) can help disadvantaged countries, such as those in Africa, benefit from an equitable distribution of resources [5].

Despite the fact that international collaboration between low-income and high-income countries was shown to be crucial for guaranteeing the transfer of technology and financial resources, national governments play an important role in promoting local healthcare. Some African countries have converted their universities and non-health industries to allow an increase in the production of and access to personal protective equipment. Another example is the partnership led by Institut Pasteur in Senegal aiming to provide a model for a COVID-19 rapid test with massive manufacturing [6].

## Conclusion

Cancer patients are known to be more vulnerable, and their care and management during the COVID-19 pandemic should be in concordance with the four principles of bioethics. Respecting those principles necessitates a close collaboration between patients, healthcare professionals, physicians, family members and ethical committees. Today, all governments should take moral clarity and realize that all lives count equally and that it is an ethical obligation to ensure that they all receive proper care.
